# Supplementary material for: Lactate dehydrogenase is the Achilles’ heel of Lyme disease bacterium Borreliella burgdorferi
Source: bioRxiv. 2025 Feb 8:2025.02.07.637162. Preprint. [Version 1] doi: 10.1101/2025.02.07.637162 (PMC11839043; doi:10.1101/2025.02.07.637162)
Supplement: Supplement 1 [file NIHPP2025.02.07.637162v1-supplement-1.pdf]

bioRxiv preprint doi: https://doi.org/10.1101/2025.02.07.637162; this version posted February 8, 2025. The copyright holder has placed this preprint (which was not certified by peer review) in the Public Domain. It is no longer restricted by copyright. Anyone can legally share, reuse, remix, or adapt this material for any purpose without crediting the original authors.

Supplementary Data

Lactate dehydrogenase is the Achilles' heel of Lyme disease bacterium *Borreliella burgdorferi*

Ching Wooen Sze[1†], Michael J Lynch[2†], Kai Zhang[1], David Neau[2], Steven Ealick[2], Brian R Crane[2*], and Chunhao Li[1,3*]

Affiliations:
[1]Department of Oral Craniofacial Molecular Biology, Virginia Commonwealth University, Richmond, Virginia, 23298
[2]Department of Chemistry and Chemical Biology, Cornell University, Ithaca, New York, 14853
[3] Department of Microbiology and Immunology, Virginia Commonwealth University, Richmond, Virginia, 23298

†These authors contribute equally: Ching Wooen Sze and Michael J Lynch
*Corresponding authors: bc69@cornell.edu; cli5@vcu.edu

Methods

Recombinant Protein Expression & Purification

The *orf* (*bb_0087*) for BbLDH was PCR amplified from wild-type B31 A3-68 using primer pair P_1/P_9 with the XhoI and SacII restriction sites engineered to the 5' and 3' end, respectively. The resultant amplicon was cloned into pGEM-Teasy vector and excised with XhoI and SacII for ligation into a modified pET100/D-TOPO expression vector with an N-terminal 10 x His 1 x FLAG tag, yielding pET-BB_0087. The resulting pET-BB_0087 plasmid was transformed into Rosetta-gami 2 (DE3) *E. coli* strain (Novagen). For protein induction, 20 ml of overnight starter culture was inoculated into 1 L of LB broth and grown to OD_{600} of 0.5 at 37°C with agitation. The expression of recombinant BbLDH was induced using 1 mM isopropyl-β-D-thiogalactoside (IPTG) at 37°C for four hours and then purified at 4°C using HisTrap HP columns following the manufacturer's instruction. A second purification step using Size Exclusion Chromatography (SEC) was added to purify the eluted protein further and dialyzed in 10 mM Tris buffer, pH 8.0 at 4°C overnight. The purified recombinant protein was aliquoted into smaller volumes and stored at -80°C for antibody production, enzymatic analysis, and crystallization. To study the significance of key residues in the function of BbLDH, pET-BB_0087 constructed above was used as template for all the site directed mutagenesis study. pET-BB_0087H178A and pET-BB_0087T146A R154A were constructed using primer pairs P_{10}/P_{11} and P_{12}/P_{13}, respectively, to establish the role of BB_0087 as a LDH. pET-BB_0087H171A was constructed using primer pair P_{14}/P_{15} to determine if BbLDH is allosterically regulated.

Production of BbLDH polyclonal antibodies

5 mg of purified recombinant wild-type BbLDH protein from above was used to generate polyclonal antibodies in rabbits on a fee-for-service basis in General Bioscience Corporation (Brisbane, CA), following a standard immunization procedure.

SEC-MALS of BbLDH with and without cofactors

bioRxiv preprint doi: https://doi.org/10.1101/2025.02.07.637162; this version posted February 8, 2025. The copyright holder has placed this preprint (which was not certified by peer review) in the Public Domain. It is no longer restricted by copyright. Anyone can legally share, reuse, remix, or adapt this material for any purpose without crediting the original authors.

BbLDH samples were prepared to a final concentration of 10 μM in MALS buffer (20 mM Tris pH 7.5, 150 mM NaCl) supplemented with 10 mM NADH, oxamate and/or NADH. Prior to injection, 10 mM β-mercaptoethanol was added to each sample and centrifuged at 13,000 rpm for 5 minutes at 4ºC. Each sample (100 mL) was injected onto a S200 (10/30, GE Lifesciences) that had been pre-equilibrated with MALS buffer for >3 CVs. The gel filtration column was coupled to a static 18-angle light scattering detector (DAWN HELEOS-II) and a refractive index detector (Optilab T-rEX) (Wyatt Technology, Goleta, CA). Data were collected every second at a flow rate of 0.7 mL/min. Data analysis was carried out using ASTRA VI, yielding the molar mass and polydispersity of the sample. Monomeric BSA was used to standardize the light scattering detector.

Table S1. Oligonucleotide primers used in this study.

Primer	Description	Sequences
**P_1**	pJSB-BB_0087FLAG (F)	5'- *CTCGAG*ATGCTTAAGTCTAATAAAGT -3'
**P_2**	pJSB-BB_0087FLAG (R)	5'- *CCGCGG*CTATTTATCATCGTCATCTTTATAATCAAATTCCATTTTATCAATATAG -3'
**P_3**	*bb_0087* IFD (left arm) (F)	5'- CGTGATATTGACTTCTCCTTCTG -3'
**P_4**	*bb_0087* IFD (left arm) (R)	5'- ACGTTTCCCGTTGAATATGGCTCATAATTACTCCATTAAATTAAAAC -3'
**P_5**	*bb_0087* IFD (right arm) (F)	5'- TTTGATGCTCGATGAGTTTTTCTAATATTAAGACCCCAAATATTTTG -3'
**P_6**	*bb_0087* IFD (right arm) (R)	5'- AATCTATTCTTCCTCCACTTAC -3'
**P_7**	*kan* cassette (F)	5'- ATGAGCCATATTCAACGGGA -3'
**P_8**	*kan* cassette (R)	5'- TTAGAAAAACTCATCGAGCA -3'
**P_9**	pET-BB_0087 (R)	5'- *CCGCCG*CTAAAATTCCATTTTATCAATAT -3'
**P_{10}**	BB_0087H178A (F)	5'- ATACATTCATATATTATGGGTGAGGCCAGTGACAGTTCTTTTGCTAC -3'
**P_{11}**	BB_0087H178A (R)	5'- ATTTTGAGTGTTCACATTAAAATGATCACTTAAAAAATATCTAAGTCTTGAAG -3'

bioRxiv preprint doi: https://doi.org/10.1101/2025.02.07.637162; this version posted February 8, 2025. The copyright holder has placed this preprint (which was not certified by peer review) in the Public Domain. It is no longer restricted by copyright. Anyone can legally share, reuse, remix, or adapt this material for any purpose without crediting the original authors.

**P_{12}**	BB_0087T146A R154A (F)	5'- GATACTTCAGCACTTAGATATTTTTTAAGTGATCA TTTTAATGTGAACACTC -3'
**P_{13}**	BB_0087T146A R154A (R)	5'- AAGAATAGTCCCAGCACCAATAACCTTATGAATA GGAAATTTGGAATATTTCATTG -3'
**P_{14}**	BB_0087H171A (F)	5'- TCAAAATATAGCGTCATATATTATGGGTGAGCAC GG -3'
**P_{15}**	BB_0087H171A (R)	5'- GTGTTCACATTAAAATGATCAC -3'

The italicized nucleotides are the engineered restriction cut sites for DNA cloning; Underlined nucleotides are FLAG tag sequence; [F] forward; [R] reverse.

Table S2: Interactions between LDH (chain A) and NADH.

Index	Interaction	Residue	Distance (Å)
1	hydrophobic	L150	3.93
2	hydrophobic	I235	3.74
3	H-bond	A12	4.0
4	H-bond	G14	3.3<br>3.1
5	H-bond	V15	3.1
6	H-bond	D37	2.6
7	H-bond	D37	2.7
8	H-bond	V38	3.5
9	H-bond	Y68	4.0
10	H-bond	G82	3.4
11	H-bond	N84	3.1
12	H-bond	N98	4.1
13	H-bond	A121	3.1
14	H-bond	N123	3.1<br>3.2

bioRxiv preprint doi: https://doi.org/10.1101/2025.02.07.637162; this version posted February 8, 2025. The copyright holder has placed this preprint (which was not certified by peer review) in the Public Domain. It is no longer restricted by copyright. Anyone can legally share, reuse, remix, or adapt this material for any purpose without crediting the original authors.

16	H-bond	G13	3.7
17	H-bond	T146	2.9
18	H-bond	water	2.9<br>2.8 x 4<br>2.7

Table S3: Interactions between LDH (chain A) and OXA.

Index	Interaction	Residue	Distance (Å)
1	H-bond	N123	2.7
2	H-bond	T231	2.8
3	H-bond	water	3.0
4	H-bond	R154	3.0
5	H-bond	R154	2.9
6	H-bond	H178	3.2

Table S4: Interactions between LDH and FBP.

Index	Interaction	Residue	Chain	Distance (Å)
1	H-bond	water	-	2.8
2	H-bond	R156	C	3.1 x 2<br>3.2
3	H-bond	H171	C	3.2 x 2<br>3.3<br>3.4
4	H-bond	N169	C	2.5
5	H-bond	Q168	C	2.6
6	H-bond	H171	A	3.2 x 2<br>3.5 x 2
7	H-bond	R156	A	3.2 x 2<br>3.4
8	H-bond	N169	A	2.6

bioRxiv preprint doi: https://doi.org/10.1101/2025.02.07.637162; this version posted February 8, 2025. The copyright holder has placed this preprint (which was not certified by peer review) in the Public Domain. It is no longer restricted by copyright. Anyone can legally share, reuse, remix, or adapt this material for any purpose without crediting the original authors.

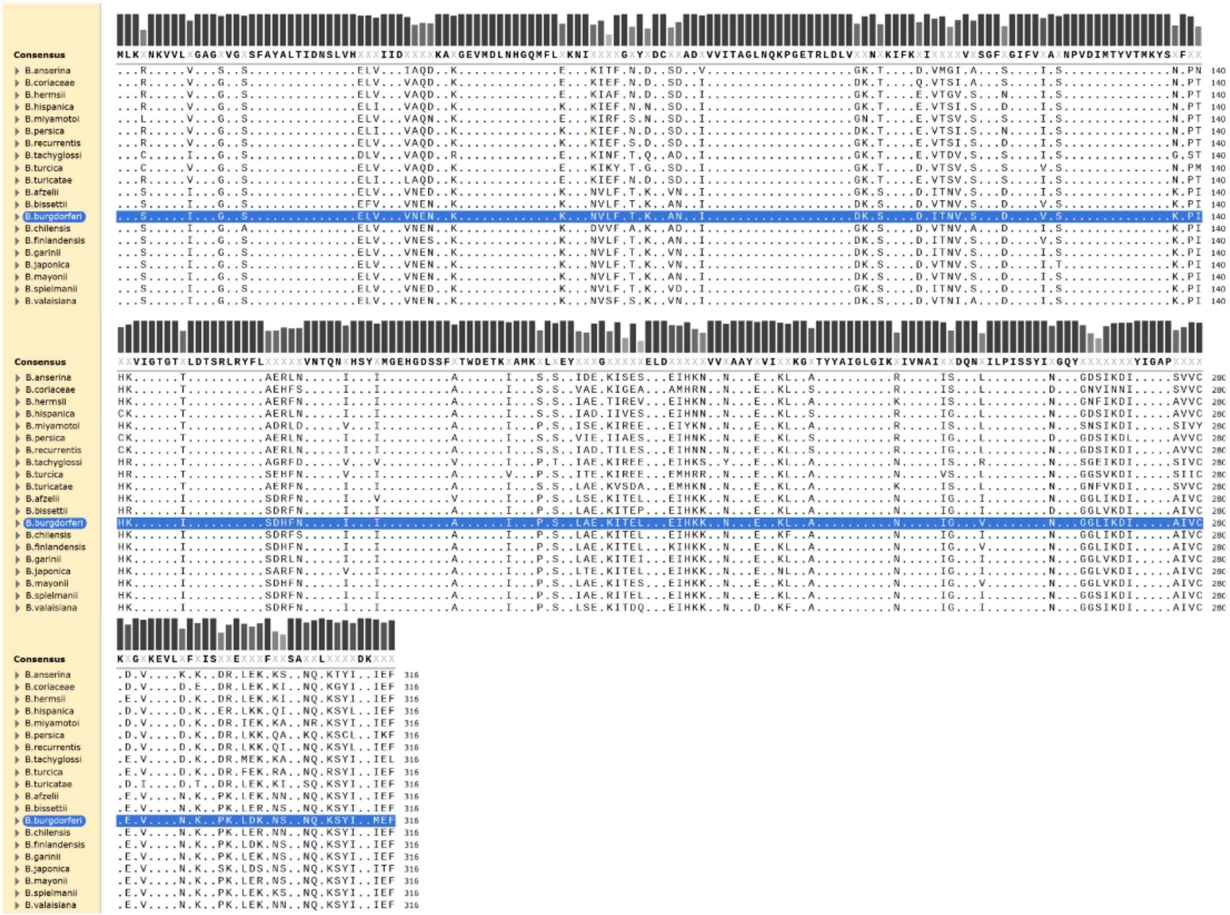

Figure S1: Sequence alignment of LDH across *Borrelia* species. LDH protein sequences for the *Borreliales* order (n = 20) were retrieved from AnnoTree (*1*), MSA generated via ClustalOmega as part of the MPI bioinformatics toolkit (*2, 3*) and visualized with SnapGene Viewer (SnapGene software (www.snapgene.com)). Sequence conservation is shown as gray bars, and a consensus sequence generated with a > 95% conserved threshold. Amino acids that are identical to the consensus sequence are indicated with a "**.**" and those that differ are denoted with their single-letter code. BbLDH sequence is highlighted for visualization purposes.

bioRxiv preprint doi: https://doi.org/10.1101/2025.02.07.637162; this version posted February 8, 2025. The copyright holder has placed this preprint (which was not certified by peer review) in the Public Domain. It is no longer restricted by copyright. Anyone can legally share, reuse, remix, or adapt this material for any purpose without crediting the original authors.

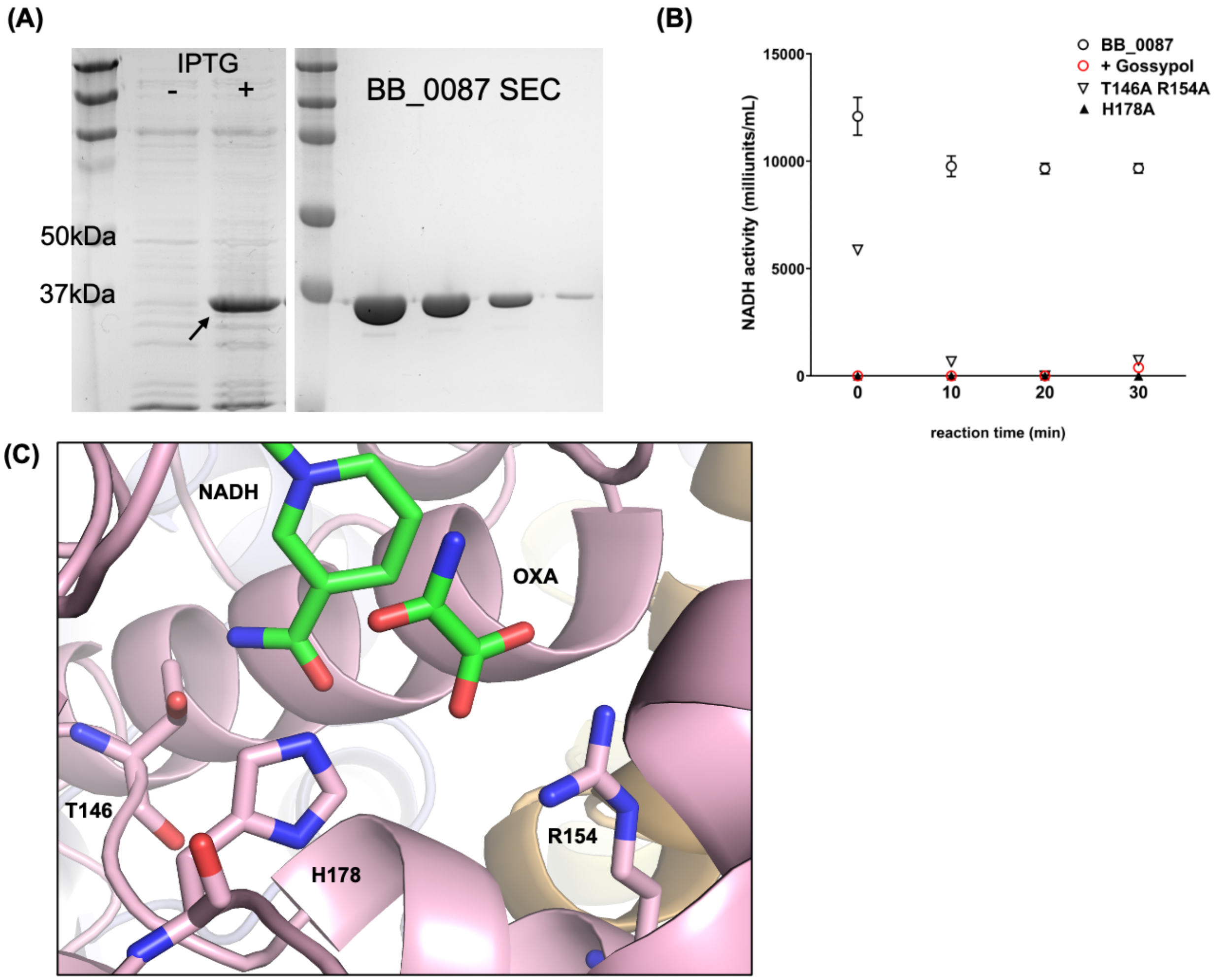

Figure S2. Expression and Purification of recombinant BB_0087. **(A)** Full length *bb_0087* sequence was cloned into a modified pET100/D-TOPO expression vector containing 10 x His and 1 x FLAG tag at the N-terminal and transformed into *E. coli* Rosetta-gami (DE3) expression cell line for induction. Upon the addition of 1 mM IPTG, induction of BB_0087 was observed around 37 kDa. Arrow indicates the induced protein. Purification of BB_0087 was performed using HiTrap His column followed by size exclusion chromatography column (SEC) to obtain homogenous protein. **(B)** BB_0087 has LDH activity. The LDH activity of purified BB_0087 was assessed using a Sigma Aldrich Lactate Dehydrogenase Assay Kit. NADH activity was detected using 50 ng of purified recombinant protein which was completely abolished upon treatment with 100 µM of gossypol. Mutation of conserved key residues (T146A R154A and H178A) found in LDH enzymes abolished the NADH activity of BB_0087. **(C)** Structure and location of conserved residues Thr-146, Arg-154 and His-178 in the NADH/OXA binding site of BbLDH. Figure was prepared using chain A, however, the location of all residues were invariant in all four chains of the BbLDH tetramer.

bioRxiv preprint doi: https://doi.org/10.1101/2025.02.07.637162; this version posted February 8, 2025. The copyright holder has placed this preprint (which was not certified by peer review) in the Public Domain. It is no longer restricted by copyright. Anyone can legally share, reuse, remix, or adapt this material for any purpose without crediting the original authors.

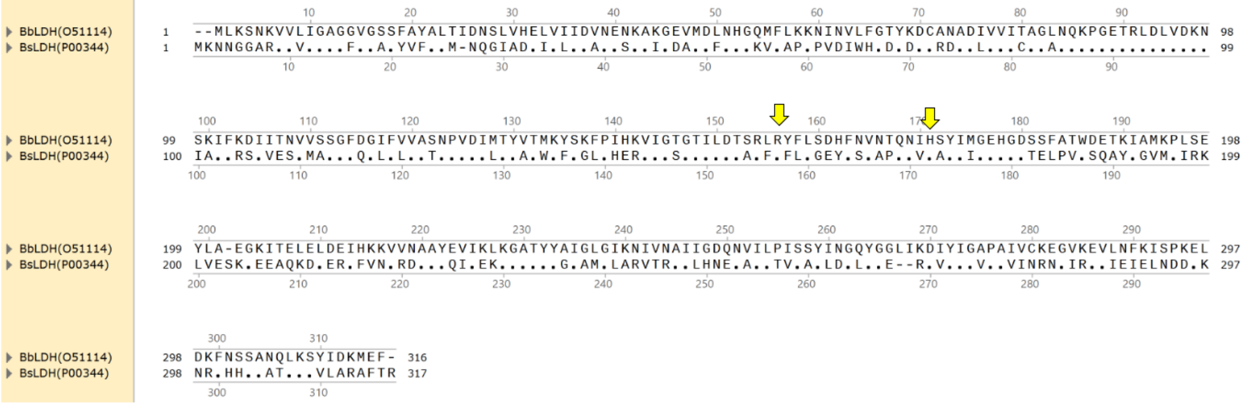

Figure S3. Sequence alignment between BbLDH (Uniprot ID: 051114) and BsLDH (Uniprot ID: P00344). Identical residues are indicated by a "." and different residues are denoted with their one-letter residue code (for Bs). Conserved FBP binding residues are marked with a yellow arrow.

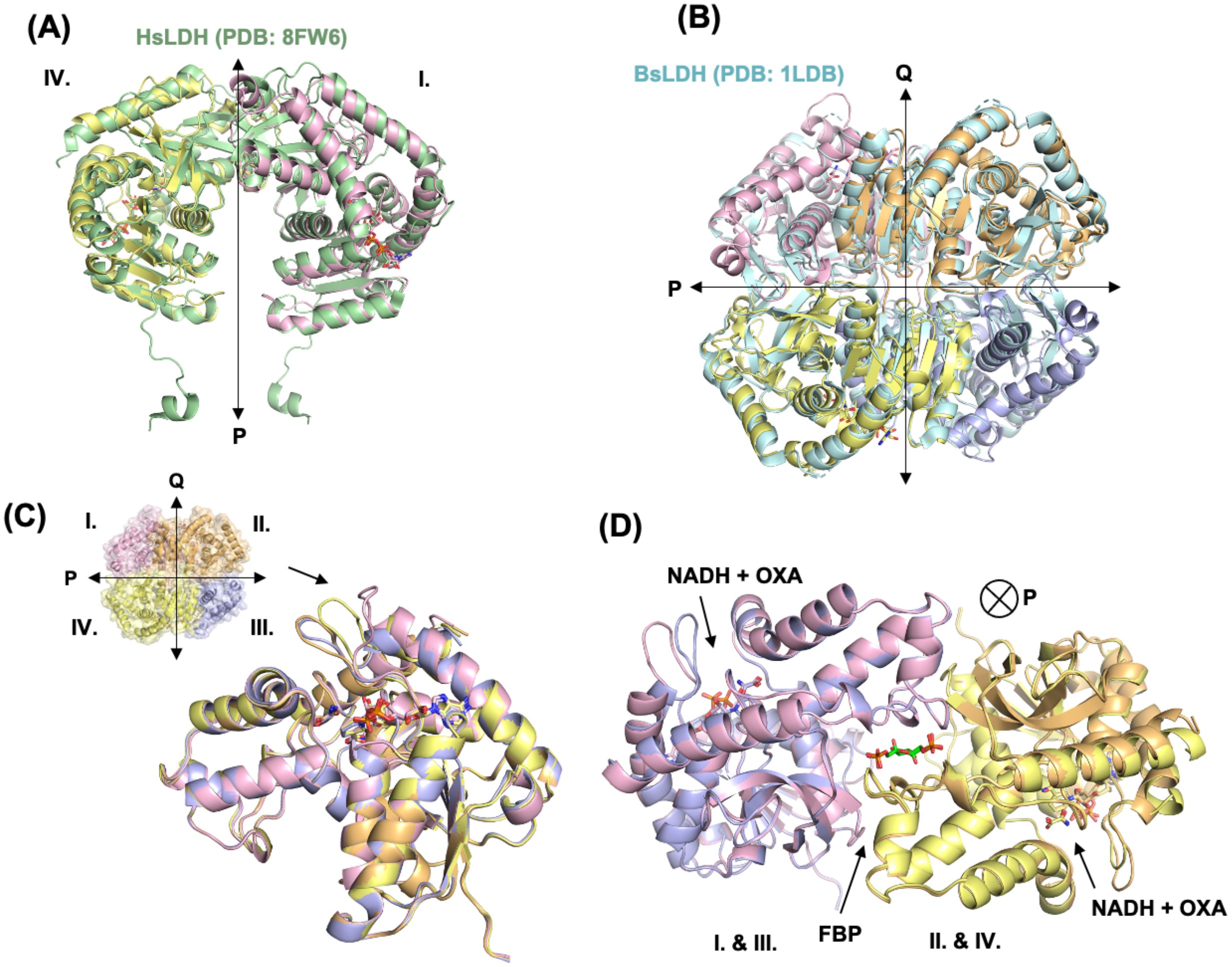

Figure S4. Structural comparison of BbLDH. **(A)** Superimposition of BbLDH dimer with HsLDH dimer (PDB 8FW6, RMSD = 1.394). **(B)** Superimposition of BbLDH and BsLDH tetramers (PDB 1LDB, RMSD = 17.270). **(C)** Structural comparison of the NADH and oxamate binding sites in each BbLDH monomer within the tetramer (RMSD range: 0.15-0.2). The major structural difference between all monomers is the relative position of the loop (Q85-R91) adjacent to the NADH/OXA

bioRxiv preprint doi: https://doi.org/10.1101/2025.02.07.637162; this version posted February 8, 2025. The copyright holder has placed this preprint (which was not certified by peer review) in the Public Domain. It is no longer restricted by copyright. Anyone can legally share, reuse, remix, or adapt this material for any purpose without crediting the original authors.

binding site (black arrow). **(D)** Structural comparison between the two FBP binding sites per BbLDH tetramer (1 FBP per dimer).

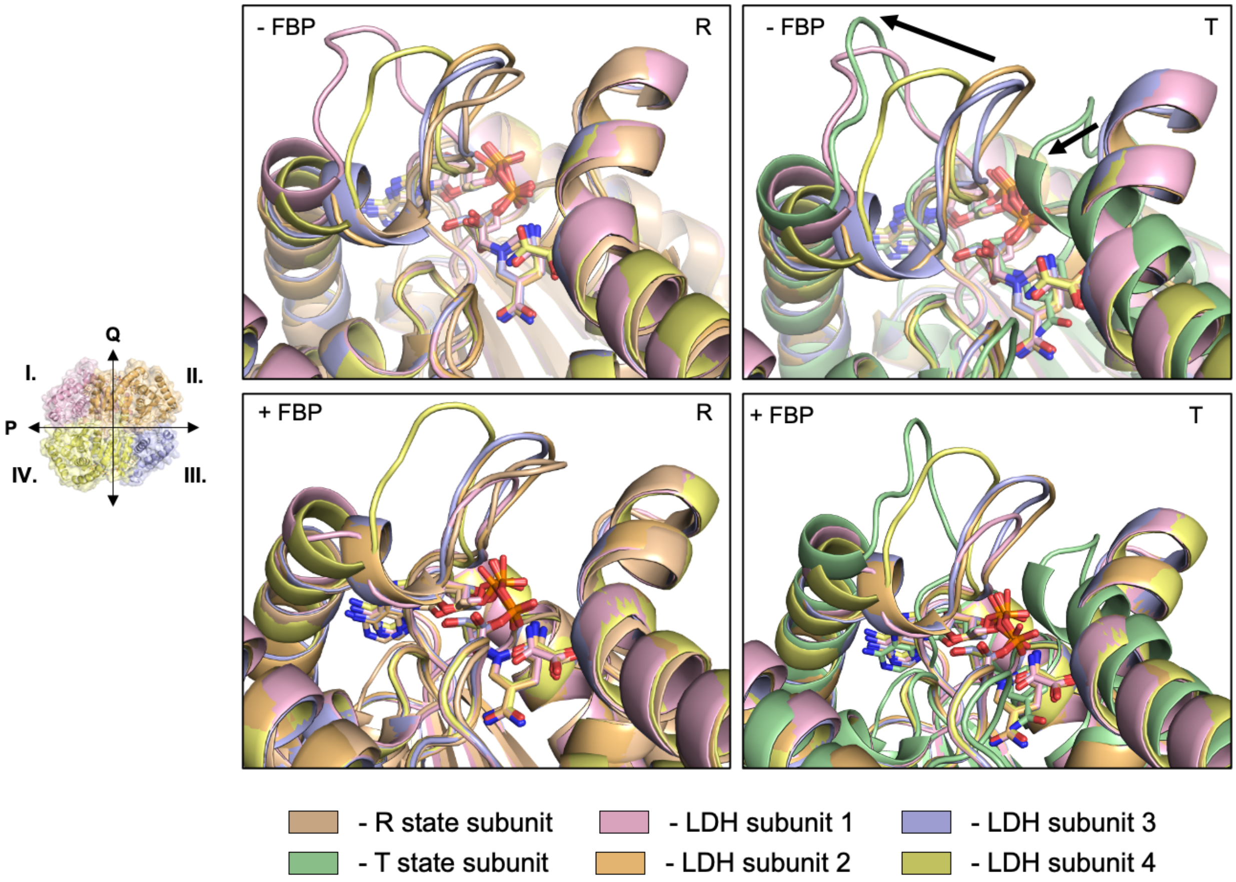

Figure S5. Structural comparison of BbLDH subunits to known R- and T-state conformations. Structural superimposition of LDH subunits from the BbLDH tetramer without (top) and with (bottom) FBP. In the left two panels *Bifidobacterium longum subsp. longum* (Bl, PDB: 1LTH) LDH subunit in the R-state (tan) is superimposed with the four BbLDH subunits, whereas in the right two panels the *B. longum* T-state subunit (green) is superimposed with the BbLDH subunits. Helix and loop motions that distinguish the T and the R states are shown with arrows on the top right.

bioRxiv preprint doi: https://doi.org/10.1101/2025.02.07.637162; this version posted February 8, 2025. The copyright holder has placed this preprint (which was not certified by peer review) in the Public Domain. It is no longer restricted by copyright. Anyone can legally share, reuse, remix, or adapt this material for any purpose without crediting the original authors.

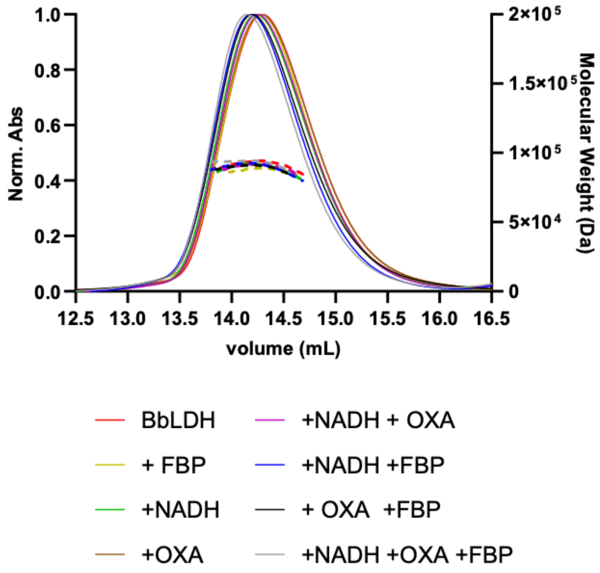

Sample	Molecular Weight (kDa)
BSA	73.2 ± 0.5%
Bb LDH	91.5 ± 0.6%
+FBP	87.1 ± 1.0%
+NADH	89.1 ± 0.6%
+OXA	89.5 ± 0.8%
+NADH +OXA	90.3 ± 0.8%
+NADH +FBP	89.5 ± 0.8%
+OXA +FBP	89.5 ± 0.9%
+NADH +OXA +FBP	93.0 ± 0.8%

Figure S6. SEC-MALS of BbLDH with and without NADH, oxamate, and FBP. (left) SEC-MALS trace of all protein samples analyzed and (right) table of the molecular weights of each sample.

bioRxiv preprint doi: https://doi.org/10.1101/2025.02.07.637162; this version posted February 8, 2025. The copyright holder has placed this preprint (which was not certified by peer review) in the Public Domain. It is no longer restricted by copyright. Anyone can legally share, reuse, remix, or adapt this material for any purpose without crediting the original authors.

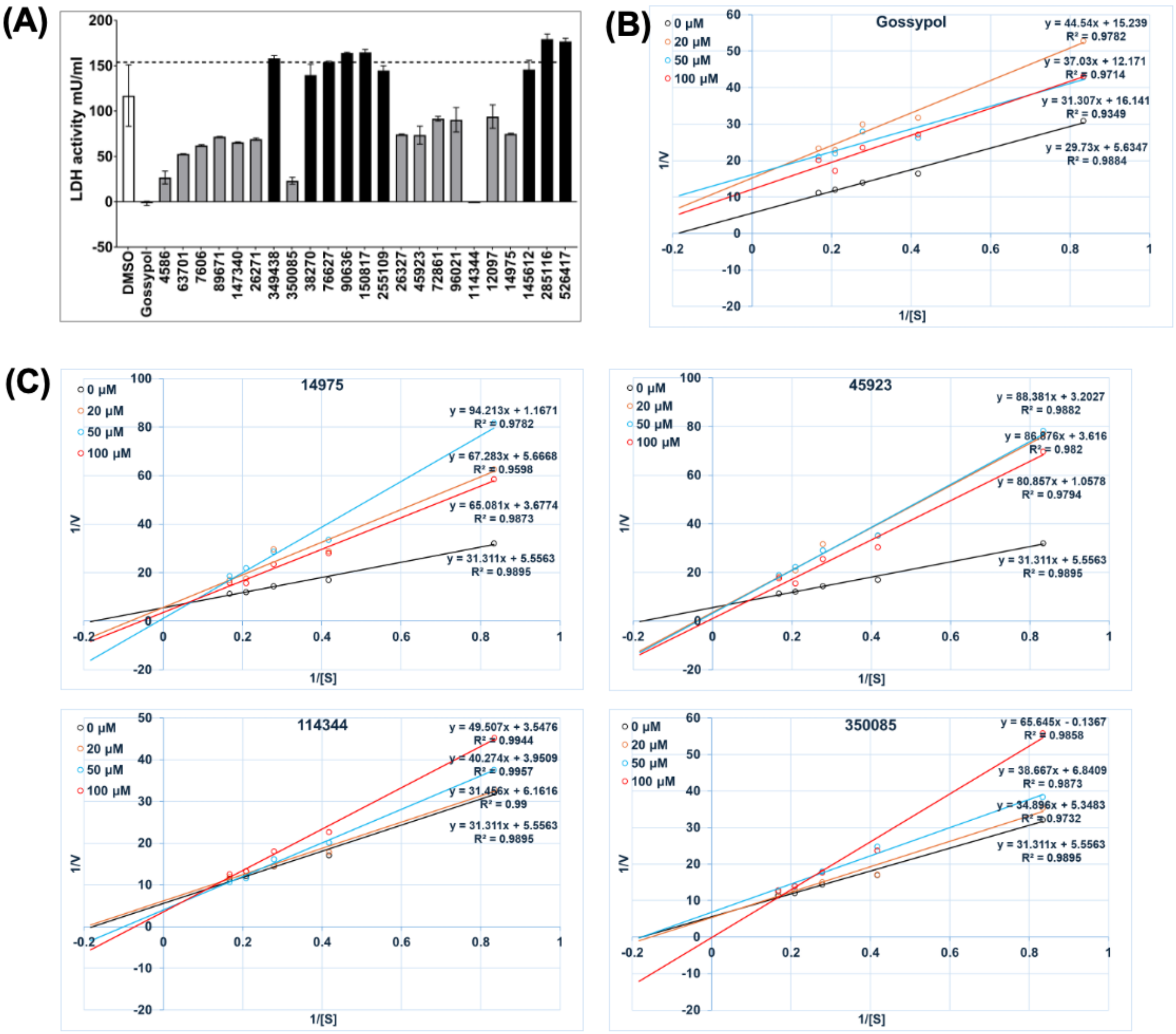

Figure S7. (A) An example of one set of screening results. DMSO was included as a vehicle control and gossypol as a positive control. Three hits (75% reduction in LDH activity as a cutoff) were identified: 350085 (270.28 Da), 45923 (216.18 Da), and 114344 (276.28 Da). Lineweaver–Burk plots of **(B)** gossypol and **(C)** 14975, 45923, 114344, and 350085 tested in this study.

bioRxiv preprint doi: https://doi.org/10.1101/2025.02.07.637162; this version posted February 8, 2025. The copyright holder has placed this preprint (which was not certified by peer review) in the Public Domain. It is no longer restricted by copyright. Anyone can legally share, reuse, remix, or adapt this material for any purpose without crediting the original authors.

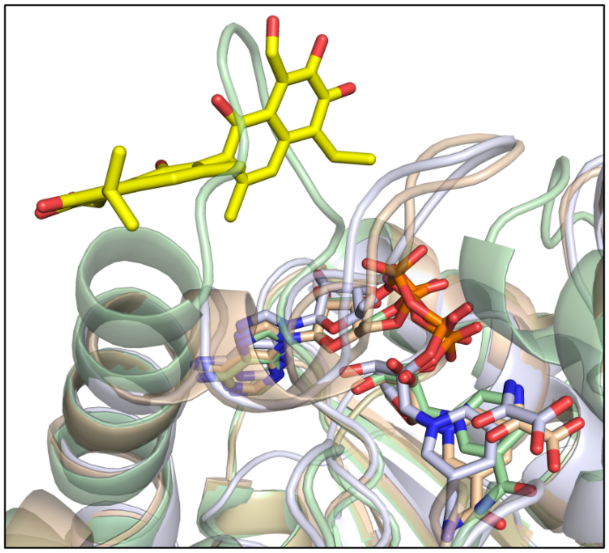

Figure S8. Gossypol docking pose with LDH model (light blue) with R- (tan) and T-state (green) BlLDH monomers (PDB: 1LTH).

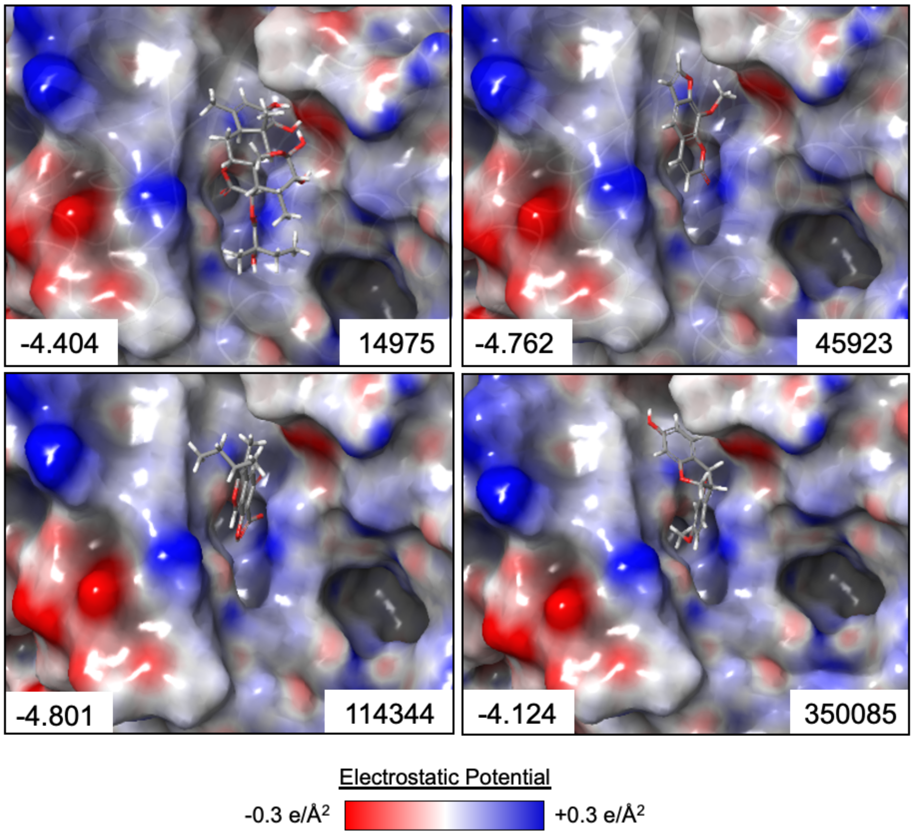

Figure S9. Top docking poses of 14975, 45923, 114344 and 350085. Top docking pose of each inhibitor to the apo BbLDH model. For each pose, the molecular surface of BbLDH is colored according to is electrostatic potential and the docking score denoted in the bottom right corner. (B)-

bioRxiv preprint doi: https://doi.org/10.1101/2025.02.07.637162; this version posted February 8, 2025. The copyright holder has placed this preprint (which was not certified by peer review) in the Public Domain. It is no longer restricted by copyright. Anyone can legally share, reuse, remix, or adapt this material for any purpose without crediting the original authors.

(E) Ligand interaction diagram showing the hydrogen bonding interactions between each inhibitor and BbLDH.

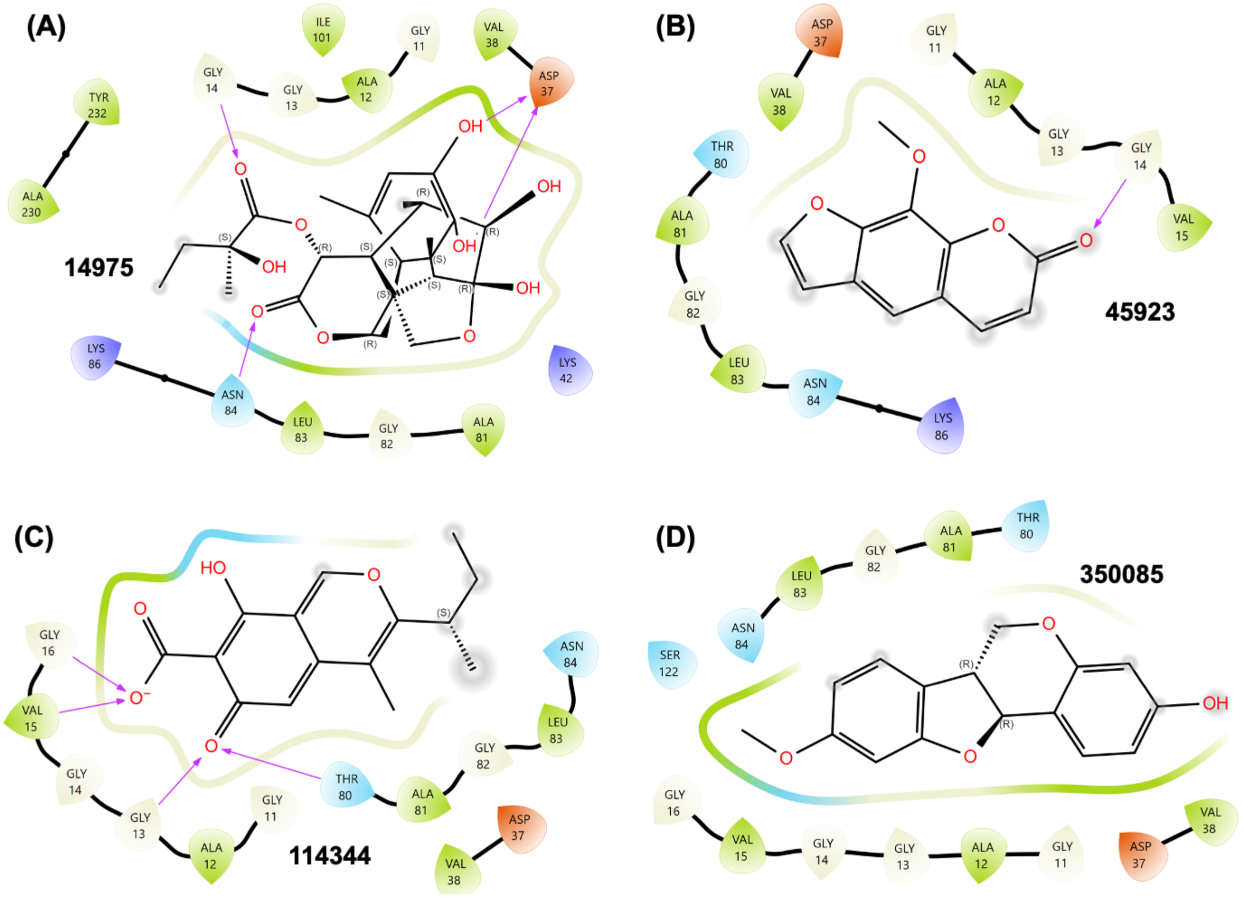

Figure S10. Interactions predicted by docking analysis. (A)-(D) Ligand interaction diagram showing the hydrogen bonding interactions between each inhibitor and BbLDH.

References and Notes

1. K. Mendler *et al.*, AnnoTree: visualization and exploration of a functionally annotated microbial tree of life. *Nucleic Acids Res* **47**, 4442-4448 (2019).
2. A. Biegert, C. Mayer, M. Remmert, J. Soding, A. N. Lupas, The MPI Bioinformatics Toolkit for protein sequence analysis. *Nucleic Acids Res* **34**, W335-339 (2006).
3. F. Madeira *et al.*, The EMBL-EBI Job Dispatcher sequence analysis tools framework in 2024. *Nucleic Acids Res* **52**, W521-W525 (2024).
